# Effects of norepinephrine and β2 receptor antagonist ICI 118,551 on whisker hair follicle mechanoreceptors dissatisfy Merkel discs being adrenergic synapses

**DOI:** 10.1186/s13041-019-0450-7

**Published:** 2019-04-03

**Authors:** Mayumi Sonekatsu, Steven Lawrence Gu, Hirosato Kanda, Jianguo G. Gu

**Affiliations:** Department of Anesthesiology and Perioperative Medicine, School of Medicine, University of Alabama at Birmingham, Birmingham, AL 35294 USA

**Keywords:** 5-hydroxytryptamine, Norepinephrine, Merkel cells, Mechanoreceptors, Touch domes, Whisker hair follicles

## Abstract

Merkel discs, located in skin touch domes and whisker hair follicles, are tactile end organs essential for environmental exploration, social interaction, and tactile discrimination. Recent studies from our group and two others have shown that mechanical stimulation excites Merkel cells via Piezo2 channel activation to subsequently activate sensory neural pathways. We have further shown that mechanical stimulation leads to the release of 5-HT from Merkel cells to synaptically transmit tactile signals to whisker afferent nerves. However, a more recent study using skin touch domes has raised the possibility that Merkel discs are adrenergic synapses. It was proposed that norepinephrine is released from Merkel cells upon mechanical stimulation to subsequently activate β2 adrenergic receptors on Merkel disc nerve endings leading to nerve impulses. In the present study, we examined effects of norepinephrine and β2 adrenergic receptor antagonist ICI 118,551 on Merkel disc mechanoreceptors in mouse whisker hair follicles. We show that norepinephrine did not directly induce impulses from Merkel disc mechanoreceptors. Furthermore, we found that ICI 118,551 at 50 μM inhibited voltage-gated Na^+^ channels and suppressed impulses of Merkel disc mechanoreceptors, but ICI 118,551 at 1 μM had no effects on the impulse. These findings challenge the hypothesis of Merkel discs being adrenergic synapses.

**Electronic supplementary material:**

The online version of this article (10.1186/s13041-019-0450-7) contains supplementary material, which is available to authorized users.

## Main

In both touch domes and whisker hair follicles, Merkel cells and their associated afferent endings form synaptic-like structures called Merkel discs [1–3]. Previous studies by our group and two others have both uncovered transduction mechanisms at Merkel discs, and also suggested that Merkel cells transmit tactile signals to their associated afferent endings via synaptic transmission [4–6]. Consistently, molecular profiling of Merkel cells have demonstrated the presence of synaptic release machineries in Merkel cells [7]. We have recently shown that Merkel discs in whisker hair follicles are serotonergic synapses [8], but a more recent study has demonstrated that Merkel discs in skin touch domes are adrenergic synapses [9]. Therefore, we attempted to determine whether key pharmacological experiments that had been used to support Merkel discs as adrenergic synapses in skin touch domes [9] could produce similar findings in whisker hair follicles.

**Fig. 1:**
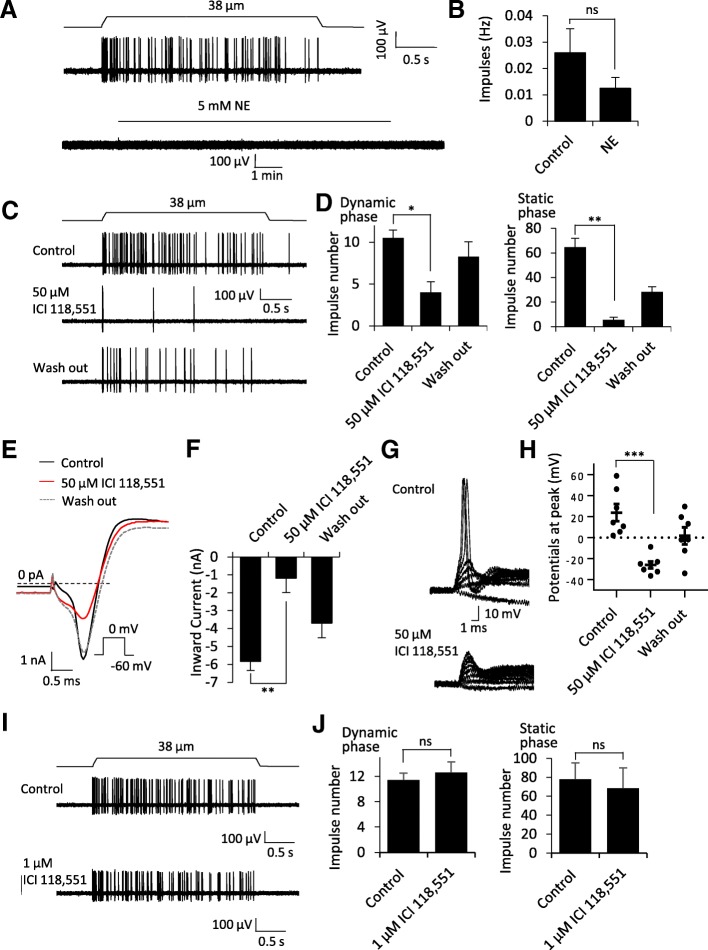
Effects of norepinephrine and β2 receptor antagonist ICI 118,551 on Merkel disc mechanoreceptors and afferent neuron excitability. **a** Top panel, sample trace shows SA1 impulses recorded from a whisker afferent fiber following mechanical displacement of its whisker hair follicle. The displacement distance was 38 μm, made by a probe at the enlargement section of the whisker hair follicle. Bottom panel, sample trace recorded from the same whisker afferent fiber in **a**, but without mechanical stimulation, in the absence and presence of 5 mM NE. **b** Summary data (*n* = 9) show the NE did not directly elicit impulses in the whisker afferent fibers displaying SA1 impulses (Merkel disc mechanoreceptors). **c** Three sample traces show SA1 impulses in the absence (control, top), presence of 50 μM ICI 118,155 (middle), and wash out of the drug (bottom). **d** Summary data of the experiment illustrated in **c** to show that 50 μM ICI 118,155 inhibited SA1 impulses in both dynamic and static phases (*n* = 4). **e** Left panel, sample traces show voltage-activated currents recorded in a trigeminal ganglion neurons in the absence (control, solid black line), presence of 50 μM ICI 118,155 (red line), and wash out of the drug (dashed line). **f** Summary data (*n* = 7) of inward currents illustrated in E in the absence (control), presence of 50 μM ICI 118,155, and wash out of the drug. **g** Two sets of sample traces show membrane responses of trigeminal neurons to currents steps in the absence (control, top panel) and presence of 50 μM ICI 118,155 (bottom). **h** Summary data (*n* = 7) of the experiment illustrated in **g** showing action potentials with peak potentials over 0 mV in control and fail of firing action potentials with peak potential below 0 mV. **i** & **j** Similar to **c** and **d** except ICI 118,155 at 1 μM was applied, which did not affect SA1 impulses (*n* = 5). In all experiments, whisker hair follicles were harvested from adult mice, perfused with Krebs bath solution. Single fiber recordings were made with a fire-polished electrode (~ 5 μm in diameter). Norepinephrine was applied for 10 min in each test, and ICI 118,551 was applied for 30 min in each test. Data represent Mean ± SEM, ns, not significantly different, *p < 0.05, **p < 0.01, ***p < 0.001, paired student’s t-test

We made recordings from individual whisker afferent nerves of mouse whisker hair follicles by using the pressure-clamped single nerve fiber recording technique. Slowly adapting type 1 (SA1) impulses could be recorded following mechanically probing whisker hair follicles (Fig. 1a, top trace), which indicated the activation of Merkel disc mechanoreceptors. The recent study has shown that Merkel disc mechanoreceptors in skin touch domes could be directly activated to induce afferent impulses by bath application of 5 mM norepinephrine (NE), a result supporting the hypothesis of Merkel discs being adrenergic synapsis [9]. We determined whether bath application of 5 mM NE could also evoke afferent impulses in Merkel disc mechanoreceptors of whisker hair follicles. However, in all Merkel disc mechanoreceptors pre-identified with evoked SA1 impulses, all of them failed to respond to the bath application of 5 mM NE for 10 to 20 min (Fig. 1a, bottom trace; Fig. 1b, *n* = 9). Thus, our results in whisker hair follicles disagreed with the recent study which showed that bath application of 5 mM NE could directly evoke afferent impulses in skin touch dome preparations [9]. In our previous study, we used rapid puff-application of a high concentration of NE and were unable to directly elicit afferent impulses in Merkel disc mechanoreceptors of whisker hair follicle preparations [8]. Thus, our previous and present results with NE dissatisfy NE as a transmitter at Merkel discs of mouse whisker hair follicles. Interestingly, bath application of 5 mM NE suppressed RA, SA1 and SA2 impulses that were evoked by mechanical stimulation in our whisker hair follicle preparations (Additional file 1: Figure S1). However, the effects on all three mechanoreceptors argue against a potential occlusion action at Merkel discs by exogenously applied NE.

We tested effects of ICI 118,551, a selective antagonist of β2 adrenergic receptor, on SA1 impulses evoked by mechanical probing to whisker hair follicles. In the recent study using skin touch dome preparations, ICI 118,551 at 50 μM was shown to suppress SA1 responses, a result that was interpreted as the involvement of β2 adrenergic receptors in tactile transmission at Merkel discs [9]. In our whisker hair follicle preparations, we also found that SA1 impulses were significantly suppressed by 50 μM ICI 118,551 (Fig. 1c&d). However, ICI 118,551 at 50 μM also significantly suppressed RA and SA2 impulses in our whisker hair follicle preparations (Additional file 1: Figure S2). The effects of 50 μM ICI 118,551 on all three mechanoreceptors led us to examine its potential non-specific effects on neuronal excitability. As shown in Fig. 1e&f with the recordings made from trigeminal ganglion neurons, voltage-gated Na^+^ inward currents were significantly inhibited by more than 80% following the application of 50 μM ICI 118,551. This was accompanied by a significant suppression of action potential firing in trigeminal neurons (Fig. 1g&h). These results strongly suggest that the inhibitory effects of ICI 118,551 at 50 μM on SA1 responses are due to its non-specific suppression of Merkel disc afferent nerve excitability. We also tested ICI 118,551 at a lower concentration of 1 μM. It has previously been shown that ICI 118,551 at concentrations of 0.01 to 1 μM selectively and significantly inhibited β2 receptors [10]. By using 1 μM, we hoped to reduce any potential non-specific effects while still substantially inhibit β2 receptors. However, impulse numbers of SA1 responses in whisker hair follicle preparations were not significantly affected following the application of 1 μM ICI 118,551 for 30 min (Fig. 1i&j). These results suggest that β2 receptors are unlikely involved in SA1 responses in the Merkel discs of whisker hair follicles.

In summary, our study presents pharmacological results that challenge the idea that Merkel discs are adrenergic synapses. This calls for the needs for more detailed studies to address the controversy about whether Merkel discs are serotonergic [8] or adrenergic synapses [9]. Future studies should also explore the possibilities that synaptic transmission mechanisms may be different between Merkel discs in whisker hair follicles and in skin touch domes [8, 9] and that co-transmitters may be used in tactile signaling at Merkel discs. Understanding the exact mechanisms of Merkel disc transmission would provide important insights into sensory physiology and pathology about the sense of touch.

## Additional file

Additional file 1: **Figure S1.** Effects of norepinephrine on rapidly RA, SA1 and SA2 impulses evoked by mechanical stimulation in whisker hair follicles. (DOCX 15 kb) A) Sample traces show RA impulses in the absence (control, top), presence of 5 mM NE (middle), and wash out of the drug (bottom). Inset in each panel shows impulses at an expanded time scale. B) Summary data (*n* = 6) of RA impulse numbers in the experiments illustrated in A. C) Sample traces show SA1 impulses in the absence (control, top), presence of 5 mM NE (middle), and wash out of the drug (bottom). D) Summary data (*n* = 5) of SA1 impulse numbers in dynamic phase (left) and static phase (right) in the experiments illustrated in C. E) Sample traces show SA2 impulses in the absence (control, top), presence of 5 mM NE (middle), and wash out of the drug (bottom). F) Summary data (*n* = 5) of SA2 impulse numbers in dynamic phase (left) and static phase (right) in the experiments illustrated in E. Impulses in each experiment was evoked by a 38-μm mechanical displacement. Norepinephrine (NE, 5 mM) was applied for 10 min in each test. Data represent Mean ± SEM, *p < 0.05, **p < 0.01, paired student’s t-test. **Figure S2.** Effects of ICI 118,551 on RA and SA2 impulses evoked by mechanical stimulation in whisker hair follicles. A) Sample traces show RA impulses in the absence (control, top), presence of 50 μM ICI 118,551 (middle), and wash out of the drug (bottom). Inset in each panel shows impulses at an expanded time scale. B) Summary data (*n* = 5) of RA impulse numbers in the experiments illustrated in A. C) Sample traces show SA2 impulses in the absence (control, top), presence of 50 μM ICI 118,551 (middle), and wash out of the drug (bottom). D) Summary data (*n* = 5) of SA2 impulse numbers in dynamic phase (left) and static phase (right) in the experiments illustrated in C. Impulses in each experiment was evoked by a 38-μm mechanical displacement. 50 μM ICI 118,551 was applied for 30 min in each test. Data represent Mean ± SEM, ns, not significantly different, **p < 0.01, ***p < 0.001, paired student’s t-test.

## Abbreviations

5-HT: 5-hydroxytryptamine
NE: Norepinephrine
RA: Rapidly adapting responses
SA1: Slowly adapting type 1 responses
SA2: Slowly adapting type 2 responses
