# Supplementary material for: Effects of norepinephrine and β2 receptor antagonist ICI 118,551 on whisker hair follicle mechanoreceptors dissatisfy Merkel discs being adrenergic synapses
Source: Mol Brain. 2019 Apr 3;12:31. doi: 10.1186/s13041-019-0450-7 (PMC6448341; doi:10.1186/s13041-019-0450-7)
Supplement: Supplementary file 1 — Figure S1. Effects of norepinephrine on rapidly RA, SA1 and SA2 impulses evoked by mechanical stimulation in whisker hair follicles. (DOCX 15 kb) A) Sample traces show RA impulses in the absence (control, top), presence of 5 mM NE (middle), and wash out of the drug (bottom). Inset in each panel shows impulses at an expanded time scale. B) Summary data (n = 6) of RA impulse numbers in the experiments illustrated in A. C) Sample traces show SA1 impulses in the absence (control, top), presence of 5 mM NE (middle), and wash out of the drug (bottom). D) Summary data (n = 5) of SA1 impulse numbers in dynamic phase (left) and static phase (right) in the experiments illustrated in C. E) Sample traces show SA2 impulses in the absence (control, top), presence of 5 mM NE (middle), and wash out of the drug (bottom). F) Summary data (n = 5) of SA2 impulse numbers in dynamic phase (left) and static phase (right) in the experiments illustrated in E. Impulses in each experiment was evoked by a 38-μm mechanical displacement. Norepinephrine (NE, 5 mM) was applied for 10 min in each test. Data represent Mean ± SEM, *p < 0.05, **p < 0.01, paired student’s t-test. Figure S2. Effects of ICI 118,551 on RA and SA2 impulses evoked by mechanical stimulation in whisker hair follicles. A) Sample traces show RA impulses in the absence (control, top), presence of 50 μM ICI 118,551 (middle), and wash out of the drug (bottom). Inset in each panel shows impulses at an expanded time scale. B) Summary data (n = 5) of RA impulse numbers in the experiments illustrated in A. C) Sample traces show SA2 impulses in the absence (control, top), presence of 50 μM ICI 118,551 (middle), and wash out of the drug (bottom). D) Summary data (n = 5) of SA2 impulse numbers in dynamic phase (left) and static phase (right) in the experiments illustrated in C. Impulses in each experiment was evoked by a 38-μm mechanical displacement. 50 μM ICI 118,551 was applied for 30 min in each test. Data represent Mean ± SEM, ns, n [file 13041_2019_450_MOESM1_ESM.docx]

1. Additional files

Additional file 1: **Figure S1**. Effects of norepinephrine on rapidly RA, SA1 and SA2 impulses evoked by mechanical stimulation in whisker hair follicles.

**A**) Sample traces show RA impulses in the absence (control, top), presence of 5 mM NE (middle), and wash out of the drug (bottom). Inset in each panel shows impulses at an expanded time scale. **B**) Summary data (n = 6) of RA impulse numbers in the experiments illustrated in **A**. **C**) Sample traces show SA1 impulses in the absence (control, top), presence of 5 mM NE (middle), and wash out of the drug (bottom). **D**) Summary data (n = 5) of SA1 impulse numbers in dynamic phase (left) and static phase (right) in the experiments illustrated in **C**. **E**) Sample traces show SA2 impulses in the absence (control, top), presence of 5 mM NE (middle), and wash out of the drug (bottom). **F**) Summary data (n = 5) of SA2 impulse numbers in dynamic phase (left) and static phase (right) in the experiments illustrated in **E**. Impulses in each experiment was evoked by a 38-µm mechanical displacement. Norepinephrine (NE, 5 mM) was applied for 10 min in each test. Data represent Mean ± SEM, **p* < 0.05, ***p* < 0.01, paired student’s t-test. **Figure S2.** Effects of ICI 118,551 on RA and SA2 impulses evoked by mechanical stimulation in whisker hair follicles. **A**) Sample traces show RA impulses in the absence (control, top), presence of 50 µM ICI 118,551 (middle), and wash out of the drug (bottom). Inset in each panel shows impulses at an expanded time scale. **B**) Summary data (n = 5) of RA impulse numbers in the experiments illustrated in **A**. **C**) Sample traces show SA2 impulses in the absence (control, top), presence of 50 µM ICI 118,551 (middle), and wash out of the drug (bottom). **D**) Summary data (n = 5) of SA2 impulse numbers in dynamic phase (left) and static phase (right) in the experiments illustrated in **C**. Impulses in each experiment was evoked by a 38-µm mechanical displacement. 50 µM ICI 118,551 was applied for 30 min in each test. Data represent Mean ± SEM, ns, not significantly different, ***p* < 0.01, ****p* < 0.001, paired student’s t-test.
